# Thermophysical Characterization of Paraffin Wax Based on Mass-Accommodation Methods Applied to a Cylindrical Thermal Energy-Storage Unit

**DOI:** 10.3390/molecules27041189

**Published:** 2022-02-10

**Authors:** Valter Silva-Nava, Ernesto M. Hernández-Cooper, Jesús Enrique Chong-Quero, José A. Otero

**Affiliations:** Tecnologico de Monterrey, Escuela de Ingeniería y Ciencias, Carr. al Lago de Guadalupe Km. 3.5, Atizapán de Zaragoza 52926, Estado de Mexico, Mexico; A00458901@itesm.mx (V.S.-N.); emcooper@tec.mx (E.M.H.-C.); jchong@tec.mx (J.E.C.-Q.)

**Keywords:** mass-accommodation methods, phase-change materials, cylindrical thermal energy-storage unit

## Abstract

Two mass-accommodation methods are proposed to describe the melting of paraffin wax used as a phase-change material in a centrally heated annular region. The two methods are presented as models where volume changes produced during the phase transition are incorporated through total mass conservation. The mass of the phase-change material is imposed as a constant, which brings an additional equation of motion. Volume changes in a cylindrical unit are pictured in two different ways. On the one hand, volume changes in the radial direction are proposed through an equation of motion where the outer radius of the cylindrical unit is promoted as a dynamical variable of motion. On the other hand, volume changes along the axial symmetry axis of the cylindrical unit are proposed through an equation of motion, where the excess volume of liquid constitutes the dynamical variable. The energy–mass balance at the liquid–solid interface is obtained according to each method of conceiving volume changes. The resulting energy–mass balance at the interface constitutes an equation of motion for the radius of the region delimited by the liquid–solid interface. Subtle differences are found between the equations of motion for the interface. The differences are consistent with mass conservation and local mass balance at the interface. Stationary states for volume changes and the radius of the region delimited by the liquid–solid interface are obtained for each mass-accommodation method. We show that the relationship between these steady states is proportional to the relationship between liquid and solid densities when the system is close to the high melting regime. Experimental tests are performed in a vertical annular region occupied by a paraffin wax. The boundary conditions used in the experimental tests produce a thin liquid layer during a melting process. The experimental results are used to characterize the phase-change material through the proposed models in this work. Finally, the thermodynamic properties of the paraffin wax are estimated by minimizing the quadratic error between the temperature readings within the phase-change material and the temperature field predicted by the proposed model.

## 1. Introduction

The thermal energy density of systems based on latent heat-storage units can be increased by using the latent heat of materials as an additional form of energy storage. The energy density of thermal energy-storage (TES) units that are only based on sensible heat is significantly lower than energy-density values achieved on latent heat thermal energy-storage (LHTES) units. The subject of solar energy harvesting in concentrating solar power plants for thermoelectric generation [1,2,3] and for domestic water heating systems [4] has become an appealing subject in research studies that are focused on the potential applications of these systems. The subject of heat storage presents one of several alternative applications that are aimed at reducing fossil fuel consumption, which has been recently representing a concerning issue due to the worldwide increase in greenhouse gas emissions. Experimental studies have been performed on several types of LHTES units to enhance heat-transfer rates and heat-storage properties. The transfer rate of thermal energy between a heat-transfer fluid (HTF) and the phase-change material (PCM) used to store energy represents a crucial parameter in TES units. The intermittence of solar energy produced by the solar irradiance oscillations during a 24 h period presents a challenging problem on backup systems based on LHTES units, due to the low thermal conductivity of PCMs used in these types of applications.

Domestic heat water applications based on PCMs have also been extensively studied. Paraffin and salt hydrates have been widely used in these kinds of applications due to their low cost and relatively high energy-storage capacity in narrow operating temperature ranges [5]. Paraffin has been used as a PCM during melting and solidification experiments in a tilted annular region, where liquid water acting as a HTF is flowing through an inner cavity [5]. The outer surface of the wall was tilted to improve the rate of heat transfer during a melting (charging) and solidification (discharge) process. Thermal energy-storage systems that combine heat-exchange strategies through copper rods and graphite particles to increase the heat-transfer rates in a cylindrical unit, have also been studied [6]. Additionally, 2D models have been used to analyse the thermal performance of truncated conical systems and cylindrical heat-storage units with fins to enhance heat transfer rates [7]. The relationship between melting and solidification times and the temperature of the HTF has been experimentally determined in a cylindrical unit, where paraffin was used as the PCM [8]. The authors determined the thermal behaviour of the PCM through temperature measurements inside the PCM. Charging times have also been experimentally obtained in a shell-and-tube LHTES unit with different ratios of the tube–shell radius [9]. The authors measured the time-dependent temperature field within the PCM, and determined the ratio of the tube–shell radius with lower melting times and higher energy densities. Numerical predictions have been validated through experimental estimations of the liquid–solid front dynamics in cylindrical systems with electrical heating by a central rod [10]. The authors determined the PCMs melting fractions and temperature variations for different electrical power values. Melting and solidification experiments on three different paraffin types were carried out in tilted cylindrical units to determine the effects of the HTF temperature and flow rate, on the thermal performance of the PCM [11].

The interest in achieving higher energy densities has led to the usage of LHTES systems. Operating temperature ranges and materials are selected according to the type of application. The thermodynamics of PCMs required to analyse LHTES devices demands more sophisticated mathematical models and numerical methods to describe the dynamics of the phase transition. Finite volume element methods have been used to describe the freezing of supercooled liquid water [12]. The authors did not consider heat transfer through natural convection and volume changes upon freezing of liquid water. Heat exchange from natural convection has been taken into account during the phase-change process in confined systems; however, density changes induced by pressure increments during the freezing of liquid water are not considered by assuming incompressible phases [13,14]. The effects of natural convection have been considered for the prediction of the melting fraction, which was experimentally estimated from temperature field measurements in cylindrical units [10,15]. The authors, however, did not consider the volume changes of the system during the phase transition, since equal densities in the liquid and solid phase were assumed. Effects on the energy stored, charging times, and melting fractions produced by considering volume changes during phase transitions at constant pressure have been addressed on planar configurations [16,17,18,19]. The authors did not take into account the effects of supercooling (superheating) and natural convection during the solidification (melting) process, despite considering high temperature gradients. Mass-accommodation methods have also been used in planar cavities where volume changes were incorporated during the freezing of liquid water [13,14].

Volume changes have been incorporated by imposing total mass as a constant of the motion, where the length of the system was promoted as a dynamical variable. The equation of motion for the system’s length guarantees that mass is not created or destroyed during the phase-change process [16]. The consistency of the obtained solutions through the additional equation of motion was verified through the behaviour of the PCM during melting and solidification in adiabatic systems. Volume changes during phase transitions in cylindrical configurations can also be taken into account through total mass conservation. Other authors have introduced an additional equation of motion for the outer radius in cylindrical and spherical geometries [20]. The motion of the external radius is governed through an adiabatic boundary condition, which would create and destroy mass during the phase change process. Two different mass-accommodation methods for cylindrical configurations are proposed in this work. One of the proposed models introduces an additional equation of motion for the outer radius of the cylindrical unit and incorporates volume changes in the radial direction. A second mass-accommodation method takes into account volume changes along the axial direction of the cylindrical unit. Melting of solid produces an excess volume of liquid that may scatter throughout the top of the storage unit, or it may be pictured as being frequently extracted from the system. Mass conservation applied along the axial direction introduces an additional equation of motion for the excess volume of liquid, according to the second method used to accommodate mass in the system. Exact steady-state solutions for the external radius and for the radius of the region delimited by the liquid–solid interface are obtained in each case. Additionally, an experimental setup is designed to analyse the temperature field within paraffin wax used as a PCM, and placed in a vertical annular region with a rigid outer wall. Finally, the second method was applied to estimate the thermodynamic parameters of the paraffin through a least-square minimization procedure.

## 2. Mass-Accommodation Methods

The system under consideration consists of an aluminium container of length *L* that constitutes an annular region with internal radius $r_{0}$ and external radius *R*. The region is occupied by a PCM and heat is transferred in a direction perpendicular to the axial symmetry axis *z*. Thermal energy is transported through a HTF that flows through a copper tube of radius $r_{0}$ along the symmetry axis *z*. The temperature at the inner wall (copper–PCM interface) of the cylindrical unit is kept constant by the HTF, and it is homogeneously distributed along the inner wall in contact with the PCM. The temperature at the outer wall (aluminium–PCM interface) is approximately constant in time and homogeneously distributed. The temperature $T_{H}$ at the copper–PCM interface is higher than the melting temperature $T_{m}$ of the PCM, and the temperature $T_{C}$ at the aluminium–PCM interface is below $T_{m}$. Thermal energy is transferred radially outwards and during the charging process, a portion of solid PCM is transformed into liquid. The homogeneous distribution of the temperature at each boundary surface guarantees that heat transfer along the *z* direction is negligible. The phase transition takes place at constant pressure and the temperature at the liquid–solid interface is constant and equal to the liquid–solid saturation temperature at thermodynamic equilibrium $T_{m}$. Therefore, superheating of solid phase during the melting process is not considered.

The temperature dependence of the thermodynamic variables is not considered. Buoyancy effects in the liquid phase are not incorporated since the temperature dependence of the liquid density is not being considered in this work. On the one hand, thermal expansion of liquid produced by temperature gradients, along with the orientation of a homogeneously heated surface relative to the gravitational field, can give rise to natural convection induced through buoyancy phenomena within the liquid [21]. On the other hand, it has been found experimentally that temperature changes in the liquid and solid phases are dominated by conduction when small liquid fractions are formed in annular regions where the heated surface is concentric to the outer surface and oriented along the axial direction [10]. The experimental results obtained in this work are used to determine the thermodynamic properties of paraffin wax at low melting fractions and when the system is in the conductive regime. Volume changes produced during the melting process due to the density difference between liquid and solid were incorporated by using two kinds of mass-accommodation methods.

### 2.1. Mass Accommodation through Radial Changes

The energy–mass balance equation at the liquid–solid interface depends on the method considered to incorporate volume changes. During melting or solidification, the system can expand or shrink in the radial direction and volume changes can be incorporated through total mass conservation. Through this method, one of the boundaries becomes a dynamic variable of motion as previously described in rectangular systems [16,22] and in confined PCMs [13,14], where incompressible phases were assumed. The energy–mass balance at the interface during a melting process in a cylindrical unit can be obtained by estimating the mass of liquid generated between *t* and t+Δt as follows:

(1) $$\frac{\Delta M_{\ell }}{\Delta t}=\frac{M_{\ell }\left(t+\Delta t\right)-M_{\ell }\left(t\right)}{\Delta t},$$

where the mass of liquid at any instant in time *t* is:

(2) $$M_{\ell }\left(t\right)=\pi \;\rho_{\ell }\;\left(\bar{r}{\left(t\right)}^{2}-r_{0}^{2}\right)\;L,$$

where $\rho_{\ell }$ is the density of the liquid phase and $\bar{r}\left(t\right)$ is the radius of the boundary constituted by the liquid–solid interface at time *t*. The net amount of thermal energy absorbed by the solid at time *t* during a melting process can be obtained through the time derivative of the previous equation, as follows:

(3) $$2\;\pi \;L\Delta h_{m}\rho_{\ell }\bar{r}\left(t\right)\frac{d\;\bar{r}\left(t\right)}{dt}=\phi \left(\bar{r}\left(t\right),t\right),$$

where $\Delta h_{m}$ is the enthalpy of formation or latent heat of fusion and ϕ(r(t),t) is the net thermal flux through the liquid–solid interface, given by:

(4) $$\phi \left(\bar{r}\left(t\right),t\right)=2\;\pi \;L\;\bar{r}\left(t\right)\left(-k_{\ell }\frac{\partial \;T_{\ell }\left(r,t\right)}{\partial \;r}{|}_{r=\bar{r}\left(t\right)}+k_{s}\frac{\partial \;T_{s}\left(r,t\right)}{\partial \;r}{|}_{r=\bar{r}\left(t\right)}\right),$$

where $k_{\ell }$($k_{s}$) is the thermal conductivity of the liquid(solid) phase and $T_{\ell }\left(r,t\right)$($T_{s}\left(r,t\right)$) is the temperature distribution in the liquid(solid) phase. Combining the last two equations, the energy–mass balance at $\bar{r}\left(t\right)$ is given by

(5) $$\rho_{\ell }\;\Delta h_{m}\;\frac{d\;\bar{r}\left(t\right)}{dt}=-k_{\ell }\frac{\partial \;T_{\ell }\left(r,t\right)}{\partial \;r}{|}_{r=\bar{r}\left(t\right)}+k_{s}\frac{\partial \;T_{s}\left(r,t\right)}{\partial \;r}{|}_{r=\bar{r}\left(t\right)}.$$

Additionally, the phase-change process can be described by estimating the mass of melted solid between *t* and t+Δt, where $\Delta M_{s}=M_{s}\left(t\right)-M_{s}\left(t+\Delta t\right)$. Using the mass of solid phase located between $\bar{r}\left(t\right)$ and R(t), an equivalent energy–mass balance at the interface can be obtained through the time derivative of $M_{s}\left(t\right)$ as follows:

(6) $$2\;\pi \;\rho_{s}\;L\;\Delta h_{m}\;\left(R\left(t\right)\;\frac{d\;R(t)}{dt}-\bar{r}\left(t\right)\;\frac{d\;\bar{r}\left(t\right)}{dt}\right)=\phi \left(r\left(t\right),t\right),$$

where R(t) is the outer radius that becomes a variable of the motion to preserve mass during the melting process. Substituting the net thermal flux at the interface given by Equation (2), an equivalent energy–mass balance at the interface can be found as follows

(7) $$\rho_{s}\;\Delta h_{m}\;\left(\frac{R(t)}{\bar{r}\left(t\right)}\frac{d\;R(t)}{dt}-\frac{d\;\bar{r}\left(t\right)}{dt}\right)=-k_{\ell }\frac{\partial \;T_{\ell }\left(r,t\right)}{\partial \;r}{|}_{r=\bar{r}\left(t\right)}+k_{s}\frac{\partial \;T_{s}\left(r,t\right)}{\partial \;r}{|}_{r=\bar{r}\left(t\right)}.$$

Equations (5) or (7) must be coupled to the differential equation obtained by imposing total mass as a constant of the motion. The outer radius R(t) becomes a dynamical variable to incorporate volume changes during the melting process. Imposing mass conservation through the time derivative of total mass, the additional equation of motion for R(t) is given by

(8) $$\rho_{\ell }\;\bar{r}\left(t\right)\;\frac{d\;\bar{r}\left(t\right)}{dt}+\rho_{s}\left(R\left(t\right)\frac{d\;R(t)}{dt}-\bar{r}\left(t\right)\;\frac{d\;\bar{r}\left(t\right)}{dt}\right)=0.$$

The time evolution of $\bar{r}\left(t\right)$ and R(t) may be described through Equations (5) and (8), or equivalently, Equations (7) and (8), where the temperature distribution at each phase is found through the local energy balance given by:

(9) $$\rho_{i}\;C_{i}\;\frac{\partial \;T_{i}\left(r,t\right)}{\partial \;t}=\frac{k_{i}}{r}\;\frac{\partial }{\partial \;r}\left(r\;\frac{\partial \;T_{i}\left(r,t\right)}{\partial \;r}\right),$$

where $C_{i}$ is the specific heat capacity of phase *i*. Additionally, it is straightforward to show that Equations (5) or (7), can also be applied when the system is subjected to boundary conditions that produce solidification of liquid phase [16].

### 2.2. Mass Accommodation through Axial Growth

**Figure 1:**
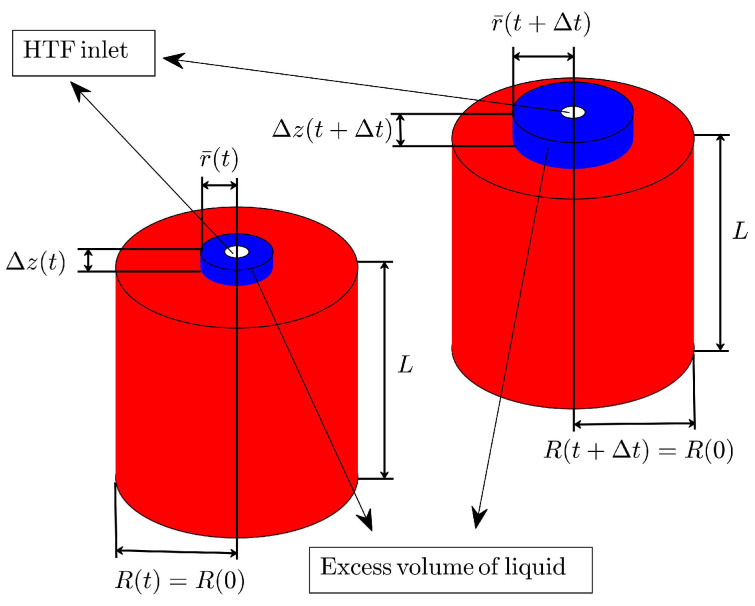
Schematic representation of liquid volume growth in the axial direction. The height of the column that represents the excess liquid at any time *t* is Δz(t). The volume of this liquid at some time *t* is $\Delta V_{\ell }\left(t\right)=\left(\bar{r}{\left(t\right)}^{2}-r_{0}^{2}\right)\left(L+\Delta z\left(t\right)\right)$ and represents the liquid that will scatter throughout the top surface of the cylinder or the volume of liquid that must be removed from the cylindrical unit.

The second method that can be used to incorporate volume changes during a melting process consists of estimating the excess volume of liquid that grows beyond the top surface and when the outer surface at r=R is constant in time. The height of the liquid column increases beyond the top surface during a melting process, given that for most PCMs the liquid density is lower than the density of the solid phase. Mass accommodation must incorporate the height of the liquid column Δz(t) as a dynamical variable instead of the outer radius *R*, as shown in Figure 1. The variable Δz(t), is related to the excess volume of liquid, as illustrated in Figure 1.

The current method promotes Δz(t) instead of the outer radius *R* as the dynamical variable of motion. The energy–mass balance at the liquid–solid interface will show subtle changes in comparison with Equation (5) when considering volume displacements along the axial symmetry axis *z*. In this scenario, the mass of liquid $M_{\ell }\left(t\right)$ at some time *t* has a slightly more complicated form than $M_{\ell }\left(t\right)$ shown through Equation (1), and is given by:

(10) $$M_{\ell }\left(t\right)=\pi \;\rho_{\ell }\;\left(\bar{r}{\left(t\right)}^{2}-r_{0}^{2}\right)(L+\Delta z\left(t\right)),$$

where Δz(t) represents the height of the excess volume of liquid, as illustrated in Figure 1. Alternatively, the expression for $M_{s}\left(t\right)$ adopts a more simpler form since the outer radius *R* is constant; then, $M_{s}\left(t\right)$ in this case is given by

(11) $$M_{s}\left(t\right)=\pi \;\rho_{s}\;L\;\left(R^{2}-\bar{r}{\left(t\right)}^{2}\right).$$

The last equation only incorporates $\bar{r}\left(t\right)$ as a dynamical variable. In this case, the energy–mass balance at the interface adopts a simpler form by considering the amount of solid transformed into liquid, instead of using the mass of liquid given by Equation (10). The mass of melted solid can be found as

(12) $$\frac{\Delta M_{s}}{\Delta t}=\frac{M_{s}\left(t\right)-M_{s}\left(t+\Delta t\right)}{\Delta t}.$$

The rate of melted solid is therefore, equal to $-d\;M_{s}\left(t\right)/dt$ in the limit when Δt→0. The rate of thermal energy absorbed at the liquid–solid interface can be obtained as:

(13) $$2\;\pi \;L\Delta h_{m}\rho_{s}\;\bar{r}\left(t\right)\frac{d\;\bar{r}\left(t\right)}{dt}=\phi \left(\bar{r}\left(t\right),t\right),$$

where $\phi (\bar{r}\left(t\right),t)$ is the net thermal flux at the interface given by Equation (4). The energy–mass balance equation is obtained by substituting the expression for $\phi (\bar{r}\left(t\right),t)$ as follows:

(14) $$\rho_{s}\;\Delta h_{m}\;\frac{d\;\bar{r}\left(t\right)}{dt}=-k_{\ell }\frac{\partial \;T_{\ell }\left(r,t\right)}{\partial \;r}{|}_{r=\bar{r}\left(t\right)}+k_{s}\frac{\partial \;T_{s}\left(r,t\right)}{\partial \;r}{|}_{r=\bar{r}\left(t\right)}.$$

which is almost identical to Equation (5); however, the solid density $\rho_{s}$ must be used instead of $\rho_{\ell }$ when considering volume changes along the axial direction. The density that must appear in the energy-mass balance equation at the interface results from the manner in which volume changes are being incorporated and is consistent with mass conservation.

The excess volume of liquid, which is related to the variable Δz(t), can be estimated by imposing total mass conservation through the time derivative of the total mass $M\left(t\right)=M_{\ell }\left(t\right)+M_{s}\left(t\right)$, as follows:

(15) $$\bar{r}\left(t\right)\frac{d\;\bar{r}\left(t\right)}{dt}\delta z\left(t\right)+\frac{1}{2}\left(\bar{r}{\left(t\right)}^{2}-r_{0}^{2}\right)\frac{d\delta z(t)}{dt}-\bar{r}\left(t\right)\frac{d\;\bar{r}\left(t\right)}{dt}\left(\frac{\rho_{s}}{\rho_{\ell }}-1\right)=0,$$

where δz(t)=Δz(t)/L represents the proportion of excess liquid and the volume of this liquid can be estimated as $\Delta V_{\ell }\left(t\right)=\pi \;L\;\delta z\left(t\right)\left(\bar{r}{\left(t\right)}^{2}-r_{0}^{2}\right)$. Equations (14) and (15) can be solved for the dynamic variables $\bar{r}\left(t\right)$ and δz(t). On the one hand, if the excess volume of liquid is being frequently removed, local heat balance at each phase may be applied through Equation (9), neglecting the effects of heat transfer produced by the liquid scattered through the top surface. On the other hand, if δz(t)≪1 during the entire charging process, heat transferred by the scattered liquid is negligible.

The phase transition can also be pictured by considering the rate of liquid mass formed during the melting process. An equivalent energy-mass balance equation at the interface can be found by considering the time derivative of the liquid mass $M_{\ell }\left(t\right)$ given by Equation (10) and using Equation (4) as follows

(16) $$\rho_{\ell }\;\Delta h_{m}\left[\frac{d\bar{r}\left(t\right)}{dt}\left(1+\delta z(t)\right)+\frac{1}{2}\left(1+{\left(\frac{r_{0}}{\bar{r}\left(t\right)}\right)}^{2}\right)\frac{d\delta z(t)}{dt}\right]=-k_{\ell }\frac{\partial \;T_{\ell }\left(r,t\right)}{\partial \;r}{|}_{r=\bar{r}\left(t\right)}+k_{s}\frac{\partial \;T_{s}\left(r,t\right)}{\partial \;r}{|}_{r=\bar{r}\left(t\right)}.$$

Conceiving the phase change in this way, the dynamical variables $\bar{r}\left(t\right)$ and δz(t) are coupled through Equation (16) and the time evolution of these variables must be obtained through the simultaneous solution of Equations (15) and (16). The system of equations $\bar{r}\left(t\right)$ and δz(t) and shown through Equations (15) and (16) is equivalent to the system given by Equations (14) and (15). Numerical solutions are easier to implement by picturing the phase-change process through Equations (14) and (15). In this situation, δz(t) is not present in Equation (14). The problem can be solved only through Equation (14), independently of the equation of motion for δz(t), as if liquid mass was being destroyed in the process. Additionally, the amount of mass destroyed or that must be constantly removed can be obtained from the solution to Equation (15) and the value of $\bar{r}\left(t\right)$.

### 2.3. Steady-State Regime

The steady-state solutions in both cases were used to verify the consistency of the numerical solutions. The steady-state solutions were used to validate the numerical method considered in this work. Although, the mathematical model where the outer radius R(t) is promoted as a dynamical variable was not used for the analysis of the experimental results, it was also solved in this section for comparison with the solutions obtained through the second mass-accommodation method.

Asymptotic time limits can be found by using the steady-state solutions to the heat equation in each phase when the system is subjected to isothermal boundary conditions. The general solution to Equation (9) in the steady-state regime is the classical logarithmic function given by:

(17) $$T_{i}^{\left(ss\right)}\left(r\right)=A_{i}\;\ln\left(r\right)+B_{i},$$

where $T_{i}^{\left(ss\right)}$ represents the temperature profile at phase *i* in the steady state (ss), and $A_{i}$, $B_{i}$ are the corresponding constants of integration. The constants shown in the last equation can be found through the boundary conditions at $r=r_{0}$, $r={\bar{r}}_{ss}$ and $r=R_{ss}$, where ${\bar{r}}_{ss}$ and $R_{ss}$ represent the steady-state values of the region delimited by the liquid–solid interface and outer radius, respectively. The system is subjected to the homogeneous isothermal boundary conditions given by:

(18) $$\begin{array}{ccc} T\left(r_{0},t\right) & = & T_{H},\\ T\left(\bar{r}\left(t\right),t\right) & = & T_{m},\\ T\left(R(t),t\right) & = & T_{C}. \end{array}$$

Applying the boundary conditions shown through Equation (18), the steady-state solution for the temperature profiles in each phase can be obtained in a straightforward manner, as follows:

(19) $$\begin{array}{ccc} T_{\ell }^{\left(ss\right)}\left(r\right) & = & -\frac{\Delta T_{H}}{\ln\left(\frac{{\bar{r}}_{ss}}{r_{0}}\right)}\ln\left(\frac{r}{r_{0}}\right)+T_{H},\\ T_{s}^{\left(ss\right)}\left(r\right) & = & -\frac{\Delta T_{C}}{\ln\left(\frac{R_{ss}}{{\bar{r}}_{ss}}\right)}\ln\left(\frac{r}{R_{ss}}\right)+T_{C}\;, \end{array}$$

where $\Delta T_{H}=T_{H}-T_{m}$ and $\Delta T_{C}=T_{m}-T_{C}$. The value of ${\bar{r}}_{s}$ can be obtained through the solution of Equation (5) in the steady state, as follows:

(20) $$k_{s}\frac{d\;T_{s}^{\left(ss\right)}\left(r\right)}{dr}{|}_{r={\bar{r}}_{ss}}=k_{\ell }\frac{d\;T_{\ell }^{\left(ss\right)}\left(r\right)}{dr}{|}_{r={\bar{r}}_{ss}}.$$

The temperature profiles in the steady state given by Equation (19) can be substituted in the last equation to obtain an expression for ${\bar{r}}_{ss}$ in terms of $R_{ss}$, which after some algebra is found, as follows

(21) $${\bar{r}}_{ss}=r_{0}{\left(\frac{R_{ss}}{r_{0}}\right)}^{\gamma }\;\;\;\;\mathrm{with}\;\;\;\;\gamma =\frac{k_{\ell }\Delta T_{H}}{k_{\ell }\Delta T_{H}+k_{s}\Delta T_{C}}.$$

Total mass conservation can be used to obtain an additional condition for ${\bar{r}}_{ss}$ and $R_{ss}$. Mass conservation in the steady state is given by:

(22) $$\rho_{\ell }\left({\bar{r}}_{ss}^{2}-r_{0}^{2}\right)+\rho_{s}\left(R_{ss}^{2}-{\bar{r}}_{ss}^{2}\right)=\rho_{\ell }\left(\bar{r}{\left(0\right)}^{2}-r_{0}^{2}\right)+\rho_{s}\left(R{\left(0\right)}^{2}-\bar{r}{\left(0\right)}^{2}\right),$$

where $\bar{r}\left(0\right)$ is the initial radius of the region delimited by the liquid–solid interface and R(0) represents the initial value of the outer radius. The right hand side of Equation (22) is the initial mass per unit length of the cylindrical unit, which should be equal to its steady-state value. Equations (21) and (22) constitute a system of nonlinear equations for ${\bar{r}}_{ss}$ and $R_{ss}$.

The solution for ${\bar{r}}_{ss}$ that corresponds to the second mass-accommodation method can be found through Equation (21), when the outer radius is constant and equal to its initial value $R_{ss}=R\left(0\right)$. However, in this method, mass conservation is pictured as illustrated in Figure 1 and Equation (22) must be changed in order to accommodate mass along the axial direction, as follows:

(23) $$\rho_{\ell }\left({\bar{r}}_{ss}^{2}-r_{0}^{2}\right)\left(1+\delta z_{ss}\right)+\rho_{s}\left(R{\left(0\right)}^{2}-{\bar{r}}_{ss}^{2}\right)=\rho_{\ell }\left(\bar{r}{\left(0\right)}^{2}-r_{0}^{2}\right)+\rho_{s}\left(R{\left(0\right)}^{2}-\bar{r}{\left(0\right)}^{2}\right),$$

The steady-state value for $\delta z_{ss}$ can be obtained from Equation (23), by substituting the solution of ${\bar{r}}_{ss}$ estimated from Equation (21) when $R_{ss}=R\left(0\right)$. The proportion of excess liquid $\delta z_{ss}$ in the steady state is then given by:

(24) $$\delta z_{ss}=\frac{\left(\rho_{s}/\rho_{\ell }-1\right)\left({\left(R\left(0\right)/r_{0}\right)}^{2\gamma }-{\left(\bar{r}\left(0\right)/r_{0}\right)}^{2}\right)}{{\left(R\left(0\right)/r_{0}\right)}^{2\gamma }-1},$$

where γ is defined through Equation (21). Finally, the proportion of scattered liquid mass $\Delta M_{\ell }^{\left(ss\right)}$ compared to the total mass of liquid in the steady state $M_{\ell }^{\left(ss\right)}$ and defined as $\delta^{\left(ss\right)}m_{\ell }=\Delta M_{\ell }^{\left(ss\right)}/M_{\ell }^{\left(ss\right)}$, is given by:

(25) $$\delta^{\left(ss\right)}m_{\ell }=\frac{\delta z_{ss}}{1+\delta z_{ss}},$$

where $\delta z_{ss}$ is given by Equation (24).

The consistency of the numerical solutions can be verified through comparison of the asymptotic time values obtained from the finite difference method, with the steady-state solutions given through Equations (21), (22), (24) and (25). Several numerical examples were performed by probing different values of γ. The relation between the steady-state values for ${\bar{r}}_{ss}$ according to each mass-accommodation method will be obtained for increasing values of γ.

A special case can be found in the limit $k_{\ell }\gg k_{s}$. The steady-state value for ${\bar{r}}_{ss}$ becomes equal to $R_{ss}$ when $k_{\ell }\gg k_{s}$, and all the solid mass is melted during the process. In this limit, where γ→1 and high melting fractions are expected, the steady-state value for $R_{ss}$ can be obtained from total mass conservation described through Equation (22) as follows

(26) $$R_{ss}^{2}=\left(1-\rho_{s}/\rho_{\ell }\right)\bar{r}{\left(0\right)}^{2}+\frac{\rho_{s}}{\rho_{\ell }}\;R^{2}\left(0\right).$$

Additionally, when the initial radius of the region delimited by the liquid–solid interface $\bar{r}\left(0\right)\ll R\left(0\right)$, the steady-state value for $R_{ss}$ at high melting fractions is approximately given by

(27) $$R_{ss}\approx \sqrt{\frac{\rho_{s}}{\rho_{\ell }}}\;R\left(0\right).$$

Equation (27) predicts the maximum possible value for $R_{ss}={\bar{r}}_{ss}$ when $k_{\ell }\gg k_{s}$. The outer radius of the second mass-accommodation method is constant, and in this limit ${\bar{r}}_{ss}=R\left(0\right)$. The relation between the steady-state values of the radii ${\bar{r}}_{ss}$ according to each mass-accommodation method in this limit is given by:

(28) $$\frac{{\bar{r}}_{ss}^{\left(1\right)}}{{\bar{r}}_{ss}^{\left(2\right)}}=\sqrt{\frac{\rho_{s}}{\rho_{\ell }}},$$

where ${\bar{r}}_{ss}^{\left(1\right)}$$\left({\bar{r}}_{ss}^{\left(2\right)}\right)$ is the radius of the region delimited by the liquid–solid interface according to the first (second) method. Equation (28) predicts the maximum possible value for the relation between ${\bar{r}}_{ss}^{\left(1\right)}$ and ${\bar{r}}_{ss}^{\left(2\right)}$ according to the mass-accommodation methods discussed in this work. Additionally, this relation is also valid for the steady-state values of the outer radius, since ${\bar{r}}_{ss}^{\left(1\right)}=R_{ss}$ and ${\bar{r}}_{ss}^{\left(2\right)}=R\left(0\right)$ when $k_{\ell }\gg k_{s}$.

Equations (27) and (28) that correspond to high melting fractions are only valid when the thermal expansion of the liquid phase is not considered or is negligible within the temperature operating range of the cylindrical unit. Equations (27) and (28) were obtained by considering liquid PCMs that lie in the conductive regime, since natural convection induced through buoyancy effects is not considered in this work. Additionally, corrections to volume changes that arise from the thermal expansion of the liquid and solid are expected. Consequently, Equations (27) and (28) are only valid when γ≪1, where γ is the dimensionless parameter defined through Equation (21), and some care must be taken when applying these equations to PCMs with high values of α or thermal units with high temperature gradients.

#### Numerical Examples

**Figure 2:**
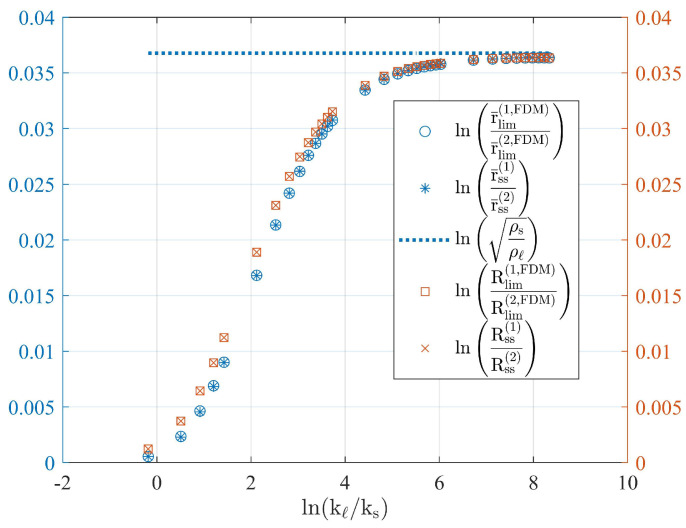
Logarithmic relation between the two mass-accommodation methods previously discussed $\ln\left({\bar{r}}^{\left(1\right)}/{\bar{r}}^{\left(2\right)}\right)$ and $\ln\left(R^{\left(1\right)}/R^{\left(2\right)}\right)$, obtained from the exact steady-state solutions given through Equations (21) and (22), and the asymptotic time values estimated through the FDM. Asterisk and cross symbols are used to represent the relation obtained through the exact steady-state values. Empty circles and squares represent the relation between the asymptotic time limits according to the numerical solutions for each mass-accommodation method. The dashed line corresponds to the predicted relation for high melting fractions according to Equation (28).

Figure 2 shows the behaviour in the steady-state regime of ${\bar{r}}_{ss}^{\left(1\right)}/{\bar{r}}_{ss}^{\left(2\right)}$ and $R_{ss}/R\left(0\right)$ for increasing values of $k_{\ell }$. The thermodynamic properties of the PCM that belong to paraffin wax are shown in Table 1 [23]. The internal radius of the cylindrical unit is $r_{0}=0.00635\;m$. The initial values of the region delimited by the liquid–solid interface and outer radius are $\bar{r}\left(0\right)=0.01\;m$ and R(0)=0.108m, respectively. Initial temperature profiles are constant and equal to $T_{\ell }\left(r,0\right)=343.15\;K$ in the liquid domain and $T_{s}\left(r,0\right)=290.15\;K$ in the solid domain. Isothermal boundary conditions are applied to the system, where the copper–PCM interface at $r=r_{0}$ is constant and equal to $T_{H}=343.15\;K$, and the outer surface at the aluminium–PCM interface r=R(t) is kept at a constant temperature value of $T_{C}=290.15\;K$.

The implicit finite difference method (FDM) [24] with a second-order approximation on the space derivatives was used to solve the mathematical model described through Equations (5), (8) and (9). Additionally, the FDM was also used to solve the model proposed through Equations (9), (14) and (15). Second-order approximations to the spacial derivatives and a first-order approximation to the time derivative were used to discretize the local energy–mass balance given by Equation (9) as follows:

(29) $$\left(\frac{\beta_{i}}{2r^{n}}-\lambda_{i}\right)T_{i}^{n-1,j}+\left(2\lambda_{i}+1\right)T_{i}^{n,j}+\left(-\frac{\beta_{i}}{2r^{n}}-\lambda_{i}\right)T_{i}^{n+1,j}=T_{i}^{n,j-1},$$

with

(30) $$\begin{array}{ccc} & & T_{i}^{n,j}=T\left(r^{n},t^{j}\right),\;T_{i}^{n,j-1}=T\left(r^{n},t^{j-1}\right), \end{array}$$

(31) $$\begin{array}{ccc} & & T_{i}^{n+1,j}=T\left(r^{n+1},t^{j}\right),\;T_{i}^{n-1,j}=T\left(r^{n-1},t^{j}\right), \end{array}$$

(32) $$\begin{array}{ccc} & & \lambda_{i}=\frac{k_{i}\Delta t}{\rho_{i}C_{i}\Delta r_{i}^{2}},\;\beta_{i}=\frac{k_{i}\Delta t}{\rho_{i}C_{i}\Delta r_{i}} \end{array}$$

where i=ℓ(s) is used for liquid (solid) phase, *n* represents the nth node, *j* the jth time level and $\Delta r_{i}$ the node separation in each phase. The dimensionless parameter at each phase $\lambda_{i}$ defined in the last equation depends on the thermodynamic properties of each phase, the node separation $\Delta r_{i}$ and the time step Δt used during the simulations. Forward and backward second-order approximations to the space derivatives that appear in Equations (5) and (14) were used as follows:

(33) $$\begin{array}{c} {\bar{r}}^{j+1}={\bar{r}}^{j}-\frac{\Delta t\;k_{\ell }}{2\rho_{i}\;\Delta r_{\ell }\;\Delta h_{m}}\left(3T_{\ell }^{n,j}-4T_{\ell }^{n-1,j}+T_{\ell }^{n-2,j}\right)+\\ \frac{\Delta t\;k_{s}}{2\rho_{i}\;\Delta r_{s}\;\Delta h_{m}}\left(-3T_{s}^{n,j}+4T_{s}^{n+1,j}-T_{s}^{n+2,j}\right), \end{array}$$

where $\rho_{i}=\rho_{\ell }$ or $\rho_{i}=\rho_{s}$ represents the density that is used in Equations (8) or (15), respectively. A first-order approximation to the time derivative of $\bar{r}$ was used with an explicit scheme to estimate the radius of the region delimited by the liquid–solid interface at the next time level ${\bar{r}}^{j+1}$.

Explicit schemes with a first-order approximation on the time derivatives were used to determine the outer radius $R^{j+1}$ or the excess liquid $\delta z^{j+1}$ at the next time level according to each mass-accommodation method. In this approximation, the outer radius $R^{j+1}$ can be obtained from the discretized form of Equation (8) and using the value of ${\bar{r}}^{j+1}$ given by the previous equation with $\rho_{i}=\rho_{\ell }$ as follows

(34) $$\left(\frac{\rho_{\ell }}{\rho_{s}}-1\right){\bar{r}}^{j}\left({\bar{r}}^{j+1}-{\bar{r}}^{j}\right)+R^{j}\left(R^{j+1}-R^{j}\right)=0.$$

The amount of excess liquid $\delta z^{j+1}$ when using the second mass-accommodation method, can be obtained from the discretized form of Equation (15) and using $\rho_{i}=\rho_{s}$ when estimating ${\bar{r}}^{j+1}$, as follows

(35) $$\left(1-\frac{\rho_{s}}{\rho_{\ell }}+\delta z^{j}\right){\bar{r}}^{j}\left({\bar{r}}^{j+1}-{\bar{r}}^{j}\right)+\frac{1}{2}\left({\left({\bar{r}}^{j}\right)}^{2}-r_{0}^{2}\right)\left(\delta z^{j+1}-\delta z^{j}\right)=0.$$

Finally, a total number of 50 nodes in the liquid layer and 100 nodes in the solid phase were used during all simulations performed in this work. The time step on each of the numerical simulations performed is Δt=0.001s.

The numerical results shown in Figure 2 correspond to the time asymptotic values for $\bar{r}\left(t\right)$ and R(t). The numerical results are compared with the steady-state values for ${\bar{r}}_{ss}$ and $R_{ss}$ given by the solution to the nonlinear system of Equations (21) and (22), and also with the steady-state value obtained through Equation (22) when the outer radius is constant and equal to R(0).

Time asymptotic values were obtained by solving each model through the FDM and for several values of $k_{\ell }$ in the range $k_{\ell }/k_{s}=\left[0.83,4166.7\right]$. Figure 2 shows the logarithmic behaviour of ${\bar{r}}_{\lim}^{\left(1,\mathrm{FDM}\right)}/{\bar{r}}_{\lim}^{\left(2,\mathrm{FDM}\right)}$ and $R_{\lim}^{\left(1,\mathrm{FDM}\right)}/R\left(0\right)$, which is consistent with the result shown through Equation (28).

## 3. Experimental Setup

**Figure 3:**
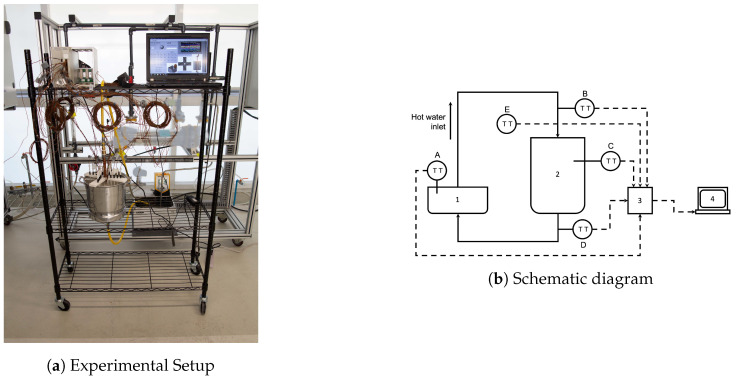
(**a**) Experimental setup with the cylindrical unit and thermocouple array. (**b**) Schematic representation of the experimental setup with the following components: 1. Lauda Thermostatic Bath, 2. Thermal energy-storage unit, 3. SCXI-1000 National Instruments module for thermocouple signal conditioning, 4. Laptop for data processing, A. 1 K-type thermocouple for heat bath temperature sensing, B. 1 K-type thermocouple for HTF inlet temperature sensing, C. 22 K-type thermocouple array for temperature sensing at the copper–PCM interface, aluminium–PCM interface and PCM temperature field estimation and D. 1 K-type thermocouple for HTF outlet temperature sensing. E. 1 K-type thermocouple for ambient temperature sensing.

The characterization of the PCM was performed through the experimental setup shown in Figure 3a. Additionally, the setup is shown schematically in Figure 3b. The components used to perform the experimental tests and the thermocouple distribution on the whole experimental array are shown schematically in Figure 3b. The experimental array consists of the following main components:

1 A 10 L cylindrical container which constitutes a vertical annular region that is used to store paraffin wax. Four arrays of thermocouples distributed in concentric circles were placed inside the PCM in its solid phase. The inner radius of the annular region is formed by a 0.5in copper tube that is placed along the axial symmetry axis of the cylindrical unit. Liquid water was gradually heated and circulated through the copper tube for thermal energy transfer at the copper–PCM interface.
2 A data acquisition system for temperature processing and data collection through the thermocouple array.
3 A system designed to control the liquid-water temperature and mass flow was developed.

**Figure 4:**
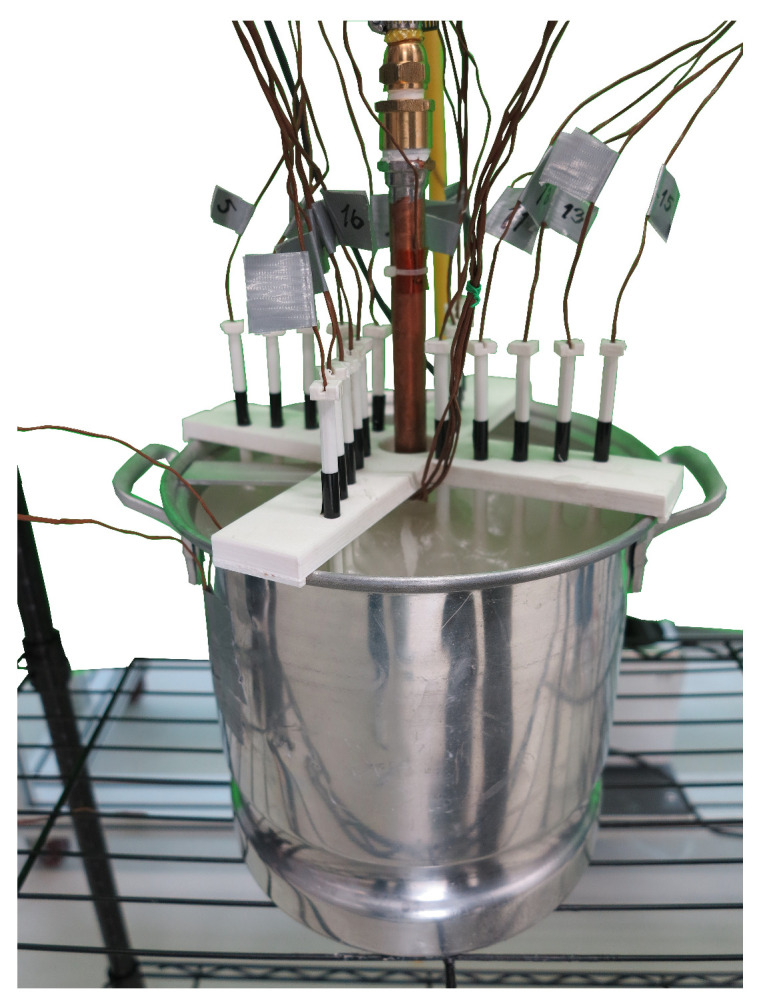
Cylindrical unit with thermocouple array for temperature sensing.

Temperature sensing at the cylindrical TES unit consists of 22 K-type thermocouples as shown in Figure 4. Thermocouples used to collect data within the PCM were distributed in four sets of concentric circles with radii $r_{1}=4.1\;\mathrm{cm}$, $r_{2}=5.8\;\mathrm{cm}$, $r_{3}=7.5\;\mathrm{cm}$ and $r_{4}=9.2\;\mathrm{cm}$. Additional thermocouples were used to measure the surrounding air temperature or ambient temperature, the copper–PCM interface temperature, the aluminium–PCM interface temperature, the inlet/outlet temperature of the liquid water, the temperature of the water at three equally spaced positions along the direction of the HTF mass flow, and the air temperature in direct contact with the aluminum shell.

**Figure 5:**
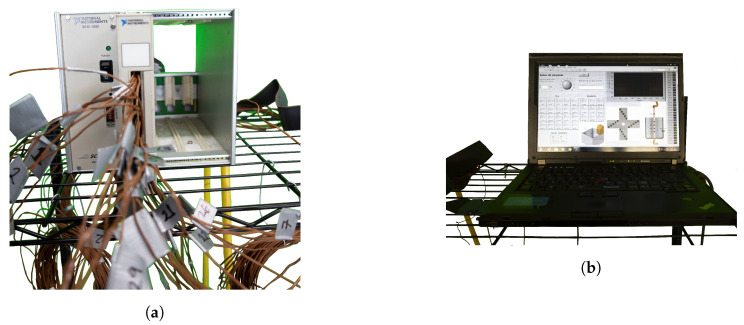
Data acquisition and processing system. (**a**) Data acquisition system. (**b**) Laptop for temperature data processing.

The data acquisition system was developed through a data collection board (National Instruments 6062E DAQ PCMCIA), a module for thermocouple signal conditioning (NI SCXI-1102B), a 32-channel isothermal terminal block (NI SCXI-1303) and a rack for instrument housing (NI SCXI-1000). The National Instruments modules are shown in Figure 5a and a laptop with a code developed in LabView for monitoring and storing data is shown in Figure 5b. The HTF (water) mass flow and temperature was controlled through a Lauda™ ECO E 10 S Heating Thermostatic Bath. Temperature data were collected with the data acquisition system just described. Constant mass flow of liquid water which acted as the HTF was fixed at 10liters/min. The HTF was gradually heated through the Lauda thermostatic bath and maximum temperature values were fixed at $70{\;}^{\circ }C$. Finally, experimental tests were carried out during a four hour period and temperature data was collected during the entire duration of the tests through the data acquisition system previously described.

## 4. Results and Discussion

The second mass-accommodation method, described in Section 2, was applied to analyse the experimental results obtained from a melting (charging) process of paraffin wax used as the PCM. The thermodynamic variables of the PCM were estimated by assuming that heat transfer within the paraffin is dominated by conduction and through the model presented in Section 2. The PCM is stored inside an annular region where water constitutes the HTF, and circulates through an inner copper tube with a radius of $r_{0}=6.35\;\mathrm{mm}$ concentric to an aluminium surface with an outer radius of R=10.8cm. Thermocouples for temperature measurements within the PCM domain were placed at a height equal to h=L/2, where L=10cm represents the total height of the heat storage unit. Thermal energy is transferred to the PCM through liquid water that circulates within the inner tube. The water acting as the HTF absorbs thermal energy from a heat bath with a thermostat fixed to $70{\;}^{\circ }C$. The outer radius is in contact with the surrounding air at ambient temperature. Liquid water is gradually heated and circulated through the inner tube from an initial temperature of $17.5{\;}^{\circ }$C until the water temperature reaches a steady-state value of $70\pm \;1{\;}^{\circ }$C. Temperature data collection within the paraffin started from the instant in which the temperature at $r=r_{0}$ reached the melting temperature $T_{m}$ of the PCM.

The temperature sensing was performed through a distribution of thermocouples in four concentric circles at fixed radii of: $r_{1}=4.1\;\mathrm{cm}$, $r_{2}=5.8\;\mathrm{cm}$, $r_{3}=7.5\;\mathrm{cm}$ and $r_{4}=9.2\;\mathrm{cm}$. Four thermocouples were placed at equal angular separations in each concentric circle, as shown in Figure 4. Therefore, the total number of thermocouples used to measure the temperature profile within the paraffin wax was 16. Each set of four sensors along the radial direction were slightly tilted, as shown in Figure 4, to minimize errors in temperature measurements due to the thermal energy absorbed by the nearest thermocouples. The temperature at each radius $r_{i}$ was estimated through the temperature average obtained from the four thermocouples distributed along each concentric circle. Additionally, one thermocouple was placed at the copper–PCM interface to estimate the temperature at $r=r_{0}$. Finally, four thermocouples were placed at the aluminium–PCM interface to determine experimental values of the temperature at r=R.

**Figure 6:**
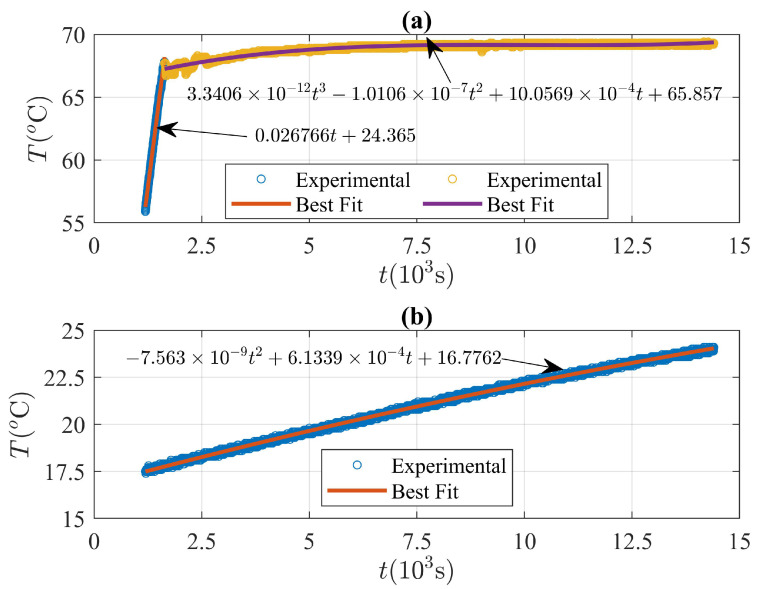
Nonhomogeneous isothermal boundary conditions. (**a**) Symbols represent time-dependent temperature values registered by the thermocouple located at the copper–PCM interface and the solid line corresponds to the best fit with the highest correlation. (**b**) Time-dependent temperature readings at the aluminium–PCM interface. Symbols represent the temperature values registered by the thermocouples located at r=0.108m and the solid line is the best fit with the highest correlation.

Figure 6 shows the average temperature as a function of time, obtained from the thermocouple readings at the copper–PCM and aluminium–PCM interface. Temperature values were registered approximately every second from the instant in which the copper–PCM interface reaches an average value equal to $T_{m}=55{\;}^{\circ }C$. The temperature at the copper–PCM interface was obtained from the experimental values shown in Figure 6 and defined as a piecewise function for the numerical simulations. Two sections were obtained and each section was approximated through a polynomial fit with the highest correlation, as illustrated in Figure 6. Additionally, the temperature at the aluminium–PCM interface for the numerical simulations was obtained through a polynomial fit with the highest correlation.

The boundary condition at the copper–PCM interface used to solve the model described in Section 2 and shown in Figure 6 is given by:

(36) $$T_{\ell }\left(r_{0},t\right)=\left\{\begin{array}{cc} 0.027t+24.37, & 1191.7\leq t\leq 1637.4\\ 3.34\times 10^{-12}\;t^{3}-1.01\times 10^{-7}\;t^{2}+10.06\times 10^{-4}\;t+65.86, & 1638.3\leq t\leq 14402\;, \end{array}\right.$$

where the temperature ranges obtained in the time domain 1191.7≤t≤1637.4 and 1638.3≤t≤14402 are $\left[56.55,68.58\right]{\;}^{\circ }$C and $\left[67.25,69.38\right]{\;}^{\circ }$C, respectively.

The root-mean-squared error (rmse) of each polynomial fit was obtained as follows:

(37) $$\mathrm{rmse}=\sqrt{\frac{1}{N}\sum_{i=1}^{N}{\left(T_{i}^{\left(\exp\right)}-T_{i}^{\left(\mathrm{fit}\right)}\right)}^{2}},$$

where *N* is the total number of observations or temperature readings and $T^{\left(\exp\right)}$($T^{\left(\mathrm{fit}\right)}$) represents the experimental (fitted) temperature values. The rmse obtained from the linear and cubic functions shown in Equation (36) is $\mathrm{rmse}=0.1804\;{}^{{\;}^{\circ }}C$ and $\mathrm{rmse}=0.1147{\;}^{\circ }$C, respectively. Additionally, the correlation coefficient $r_{c}^{2}$ was determined from several polynomial fits, and the function with the highest value of $r_{c}^{2}$ is shown in Equation (36). The correlation coefficient was obtained through the following relation:

(38) $$r_{c}^{2}=1-\frac{\sum_{i=1}^{N}{\left(T_{i}^{\left(\exp\right)}-T_{i}^{\left(\mathrm{fit}\right)}\right)}^{2}}{\sum_{i=1}^{N}{\left(T_{i}^{\left(\exp\right)}-T_{\mathrm{avg}}^{\left(\exp\right)}\right)}^{2}},$$

where $T_{\mathrm{avg}}^{\left(\exp\right)}$($T_{\mathrm{avg}}^{\left(\mathrm{fit}\right)}$) is the average temperature of the experimental (fitted) data. The highest correlation coefficient found corresponds to a linear and cubic function where $r_{c}^{2}=0.9973$ and $r_{c}^{2}=0.9495$, respectively.

Finally, the boundary condition at the aluminium–PCM interface was determined from the experimental data shown in Figure 6 as follows:

(39) $$T_{s}\left(R(t),t\right)=-7.56\times 10^{-9}\;t^{2}+6.13\times 10^{-4}\;t+16.78\;\mathrm{for}\;1191.7\leq t\leq 14402,$$

where the temperature range in the time domain given by the above equation is $\left[17.50,24.04\right]{\;}^{\circ }$C as illustrated in Figure 6. The polynomial shown in the last equation corresponds to the function with the highest correlation of $r_{c}^{2}=0.9985$ and a root-mean-squared error of $\mathrm{rmse}=0.0731\;{\;}^{\circ }$C.

**Figure 7:**
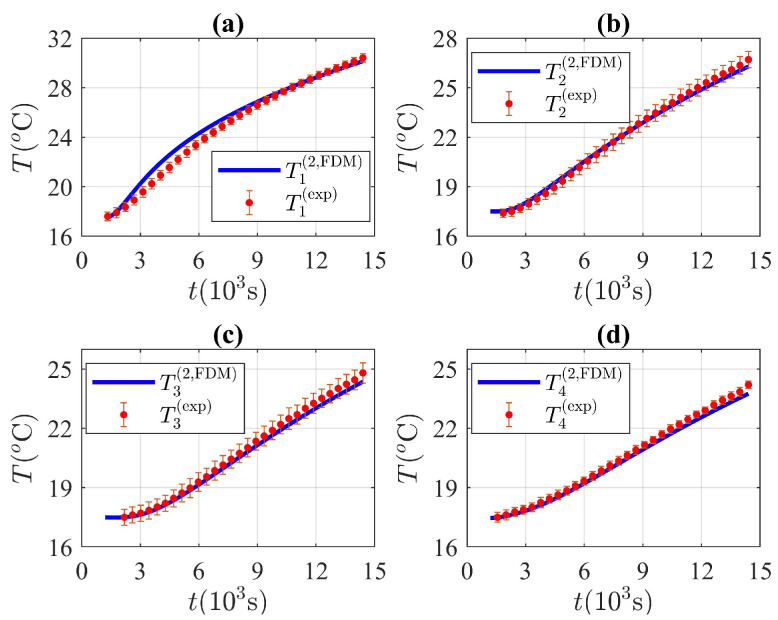
Time evolution of the temperature at each thermocouple radial position according to the the experimental and numerical results. Experimental values of the average temperature at each radial coordinate (**a**) $r_{1}=4.1\;\mathrm{cm}$, (**b**) $r_{2}=5.8\;\mathrm{cm}$, (**c**) $r_{3}=7.5\;\mathrm{cm}$ and (**d**) $r_{4}=9.2\;\mathrm{cm}$, respectively are shown in red circles and the result obtained through the FDM is shown in solid lines. The numerical result was obtained through the solution of the model described by Equations (9), (14) and (15) and through the set of thermodynamic variables $k_{\ell },\;k_{s},\;C_{\ell }\;\;\mathrm{and}\;\;C_{s}$ with the lowest quadratic error shown by Equation (40).

The temperature at each value of $r_{i}$ considered in the experimental setup shown in Figure 4 was obtained through the temperature-averaged readings, registered by the set of thermocouples distributed along each concentric circle. The average temperature at each radius was registered at equally spaced time intervals, as shown in Figure 7.

Liquid and solid samples of paraffin wax were prepared to estimate the liquid and solid densities. The density of liquid paraffin was determined by pouring a fixed volume of PCM on a beaker previously placed on a scale. Four samples of liquid PCM with different volumes at $70{\;}^{\circ }$C were used to estimate the density of the liquid phase, and an average density of $\rho_{\ell }=735.25\;\mathrm{kg}/m^{3}$ was estimated. Similarly, four solid cylindrical samples of PCM with different volumes at $20{\;}^{\circ }$C were used to determine the density of the solid phase. The volume and mass of each solid cylinder was measured and an average density of solid PCM $\rho_{s}=849.39\;\mathrm{kg}/m^{3}$ was estimated. The melting temperature $T_{m}=55{\;}^{\circ }$C was determined through the liquid–solid coexistence of the paraffin wax close to thermodynamic equilibrium.

Thermal conductivities and specific heat capacities were estimated through the second mass-accommodation method described in Section 2 and using the nonhomogeneous isothermal boundary conditions given by Equations (36) and (39). The temperature dependence of the thermodynamic variables in the operating temperature range of the cylindrical unit of $\left[17,70\right]{\;}^{\circ }$C was not considered. The latent heat of fusion can be approximated as $\Delta h_{m}=\left(C_{\ell }-C_{s}\right)\;T_{m}$ assuming that $C_{\ell }$ and $C_{s}$ are close to their saturation values at $T_{m}=55{\;}^{\circ }C$. The mass-accommodation method described through Equations (14) and (15) for the dynamical variables $\bar{r}\left(t\right)$ and δz(t), and the local energy balance at each phase given by Equation (9) was implemented through the implicit FDM previously described. The initial radius of the region delimited by the liquid–solid interface $\bar{r}\left(0\right)$ was very close to the radius of the inner tube, since data collection started very close to the melting temperature of the PCM. A value of $\bar{r}\left(0\right)=6.36\;\mathrm{mm}$ was used and a temperature of $55{\;}^{\circ }$C was established at each node within the initial liquid layer. The HTF was circulated at ambient temperature and gradually heated before data collection, until the copper–PCM interface reached the melting temperature of the PCM. During this previous stage, the PCM was found in its solid state and a logarithmic temperature profile was registered by the thermocouples when data acquisition started. The logarithmic temperature distribution obtained from the gradual heating of the solid PCM was used as the initial temperature profile in the solid phase. Low melting fractions are expected in the range of thermal conductivities considered during the analysis of experimental data and according to the models introduced in this work.

Figure 7 shows the results obtained for the temperature at each concentric circle of radius $r_{i}$. The time evolution of the average temperature value at each radial coordinate $r_{i}$ is shown in Figure 7. The FDM solutions to the model previously described are also shown in solid lines. The numerical solutions were obtained for several possible values of $k_{\ell }$, $k_{s}$, $C_{\ell }$ and $C_{s}$. The numerical results were compared with the experimental temperature values shown in Figure 7, and a quadratic error function was defined to find the set of thermodynamical parameters that best reproduce the experimental data. The quadratic error used was defined as follows:

(40) $$E_{T}\left(k_{\ell },k_{s},C_{\ell },C_{s}\right)=\sum_{j=1}^{n}\;{\left(T_{i}^{\left(\exp\right)}\left(t_{j}\right)-T_{i}^{\left(FDM\right)}\left(t_{j}\right)\right)}^{2},$$

where $T_{i}^{\left(\exp\right)}\left(t_{j}\right)$ represents the average temperature registered by the ith thermocouple and $T_{i}^{\left(FDM\right)}\left(t_{j}\right)$ is the temperature obtained through the numerical solution of the second mass-accommodation method at each time value $t_{j}$ shown in Figure 7 and at each thermocouple radial position $r_{i}$. The error function is evaluated for each particular set of thermodynamic parameters $k_{\ell },k_{s},C_{\ell },C_{s}$, where a total of $10^{4}$ sets of thermodynamic parameters was investigated in a range of possible values to find the least square error. The result is illustrated in Figure 7, where the average temperatures registered at each sensor are shown in red circles. The error bars correspond to the standard deviation obtained from the temperature readings registered by each of the four sensors distributed along a particular concentric circle of radius $r_{i}$. Figure 7 also shows the numerical result obtained with the set of thermodynamic parameters that minimize the error defined through Equation (40). The numerical results shown in Figure 7 correspond to the solutions of the second mass-accommodation method described previously and defined through Equations (9), (14) and (15).

Collected temperature values $T_{i}^{\left(\exp\right)}$ through the thermocouple data acquisition system are shown in Figure 7 at the radial coordinates $r_{1}=4.1\;\mathrm{cm}$, $r_{2}=5.8\;\mathrm{cm}$, $r_{3}=7.5\;\mathrm{cm}$ and $r_{4}=9.2\;\mathrm{cm}$. The solutions obtained with the FDM and according to the second mass-accommodation method $T_{i}^{\left(2,\mathrm{FDM}\right)}$ constitute the numerical solutions with the set of thermodynamic parameters shown in Table 2 that minimize the error given by Equation (39). According to the definition of the latent heat as the difference between liquid and solid enthalpies at the saturation temperature, and according to the specific heat capacities shown in Table 2, the latent heat of fusion of the paraffin wax estimated in this work is $\Delta h_{m}=206.73\;\mathrm{kJ}/\mathrm{kg}$.

**Figure 8:**
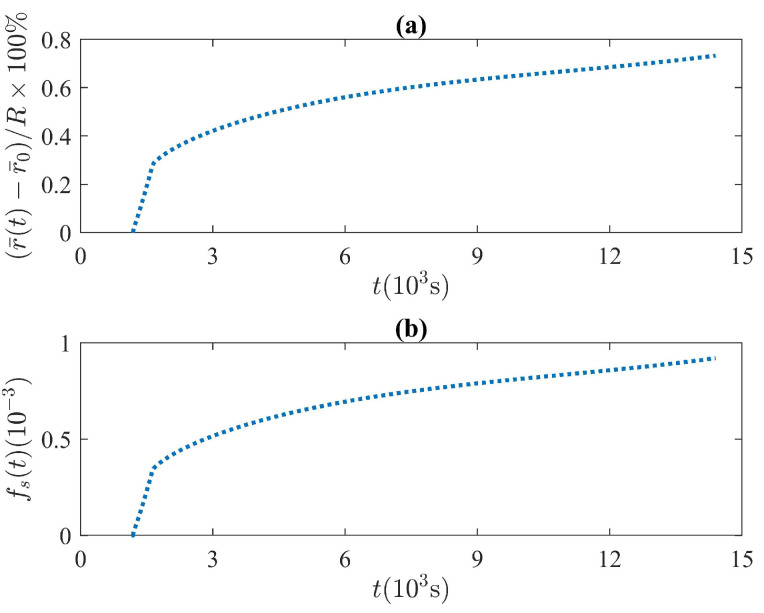
(**a**) Numerical solution to the liquid–solid interface motion. The results illustrate the relation between the liquid’s thickness and the outer radius of the thermal unit, according to the numerical solution of the proposed model with the set of thermodynamic variables shown in Table 2. (**b**) Time evolution of $f_{s}$ obtained from the numerical solutions to the proposed model and with the set of parameters shown in Table 2.

According to the second mass-accommodation model and the type of boundary conditions applied during the experimental tests, small values of $\bar{r}\left(t\right)\ll \;R\left(0\right)$ are expected since ${\bar{r}}_{ss}\ll \;R\left(0\right)$ when $k_{\ell }\ll \;k_{s}$. Close to this limit, when γ≪1, and according to Equation (21), the radial displacement of the liquid–solid interface is very small compared to the outer radius as shown in Figure 8. The time evolution of the fraction of melted solid $f_{s}=\left(M_{s}\left(0\right)-M_{s}\left(t\right)\right)/M_{s}\left(0\right)$ was obtained during the time domain shown in Figure 8. The fraction of melted solid was estimated through the set of thermodynamic variables that minimizes the quadratic error given by Equation (40) and shown in Table 2. The results illustrated in Figure 8 show that heat transfer within the liquid phase lies in the conductive regime where a small thickness of liquid PCM layer is formed, and very small fractions of melted solid $f_{s}\approx \;10^{-3}$ are observed.

## 5. Conclusions

In this work, two mass-accommodation methods that consider volume changes during PCM melting in a vertical annular region were proposed. Mass conservation introduces an additional equation of motion that depends on the way in which mass is distributed. On the one hand, the outer radius was promoted as a dynamical variable to accommodate mass in the radial direction. On the other hand, the excess volume of liquid becomes a dynamical variable that is used to accommodate mass in the axial direction. We also found that the local energy–mass balance at the liquid–solid interface depends on the particular mass-accommodation method being used. Exact analytical expressions were found for the steady-state values in each case. Additionally, the relationship between the dynamical variables in the steady state and for high melting rates is proportional to the relationship between liquid and solid densities. The numerical solutions were verified through the steady-state solutions found in this work and for increasing values of $k_{\ell }$. The thermodynamic properties of a paraffin were estimated through a minimization process. Experimental measurements of the temperature field within the PCM were used to characterize the behaviour of the PCM at low melting rates. A series of numerical simulations with different thermodynamic parameter values were performed to find the set of thermodynamic variables that minimizes the error between the experimental and numerical results. Finally, we confirm that conduction constitutes the main heat-transfer mechanism in cylindrical units with low melting rates. 

## Figures and Tables

**Table 1:** Thermodynamic properties and liquid–solid saturation values of paraffin wax according to the authors of Ref. [23].

$T_{m}$	$\Delta h_{m}$	$k_{\ell }$($k_{s}$)	$C_{\ell }$($C_{s}$)	$\rho_{\ell }$($\rho_{s}$)
K	(kJ/kg)	(W/m·K)	(kJ/kg·K)	($\mathrm{kg}/m^{3}$)
317	266.0	0.24 (0.24)	2.95 (2.51)	760 (818)

**Table 2:** Thermodynamic properties and liquid–solid saturation values of paraffin wax estimated through the experimental measurements and minimization of the quadratic error defined in this work.

$T_{m}$	$\Delta h_{m}$	$k_{\ell }$($k_{s}$)	$C_{\ell }$($C_{s}$)	$\rho_{\ell }$($\rho_{s}$)
K	(kJ/kg)	(W/m·K)	(kJ/kg·K)	($\mathrm{kg}/m^{3}$)
328	206.73	0.05(0.317)	3.20(2.57)	760(818)

## Data Availability

The data presented in this study are available on request from the corresponding author.

## Abbreviations

The following abbreviations and symbols are used in this manuscript:

CSP	Concentrating Solar Power
PCM	Phase-change material
TES	Thermal energy storage
LHTES	Latent heat thermal energy storage
HTF	Heat-transfer fluid
FDM	Finite difference method
rmse	Root-mean-squared error
$k_{\ell }$	Thermal conductivity of the liquid
$k_{s}$	Thermal conductivity of the solid
$C_{\ell }$	Specific heat capacity of the liquid
$C_{s}$	Specific heat capacity of the solid
$\rho_{\ell }$	Liquid density
$\rho_{s}$	Solid density
$\Delta h_{m}$	Latent heat of fusion
$T_{m}$	Melting temperature
*L*	Height of cylindrical unit
*R*	Outer radius
$r_{0}$	Copper tube radius
$\bar{r}$	Radius of region delimited by the liquid–solid interface
$R_{ss}$	Steady state of outer radius
${\bar{r}}_{ss}$	Steady-state value of $\bar{r}$
$T_{\ell }\left(r_{0},t\right)$	Temperature at the copper–PCM interface
$T_{s}\left(R,t\right)$	Temperature at the aluminium–PCM interface
γ	Dimensionless exponent
$r_{c}$	Correlation coefficient
$f_{s}$	Fraction of melted solid
